# Effect of shift work on fatigue and sleep in neonatal registrars

**DOI:** 10.1371/journal.pone.0245428

**Published:** 2021-01-14

**Authors:** Ajay P. Anvekar, Elizabeth A. Nathan, Dorota A. Doherty, Sanjay K. Patole

**Affiliations:** 1 Department of Neonatal Paediatrics, Perth Children Hospital, Perth, Australia; 2 Women and Infants Research Foundation, KEM Hospital for Women, Perth, Australia; 3 Division of Obstetrics and Gynaecology, School of Medicine, University of Western Australia, Perth, Australia; 4 Department of Neonatal Paediatrics, King Edward Memorial Hospital, Perth, Western Australia, Australia; 5 School of Medicine, University of Western Australia, Perth, Western Australia, Australia; University of Wuerzburg, GERMANY

## Abstract

**Objective:**

We aimed to study fatigue and sleep in registrars working 12-hour rotating shifts in our tertiary neonatal intensive unit.

**Methods and participants:**

This study involved neonatal registrar’s working day (08:00–21:00) and night (20:30–08:30) shifts. Participants maintained a sleep diary, answered a self-reported sleepiness questionnaire assessing subjective sleepiness, and performed a 10-minute psychomotor vigilance task (PVT) at the start and end of each shift. Primary outcomes: (1) Fatigue at the (i) “start vs end” of day and night shifts, (ii) end of the “day vs night” shifts, and (iii) end of “first vs last shift” in block of day and night shifts. (2) Duration and quality of sleep before the “day vs night” shifts. Mean reaction time (RTM), relative coefficient of variation (RTCV), and lapses (reaction time > 500ms) were used as measures of fatigue on PVT. Secondary outcome: Subjective sleepiness (self-reported sleepiness questionnaire) at the ‘start vs end” of day and night shifts.

**Results:**

Fifteen registrars completed the study. Acuity was comparable for all shifts. (1) Psychomotor responses were impaired at the end vs start of day shifts [RTM (p = 0.014), lapses (p = 0.001)], end vs start of night shifts [RTM (p = 0.007), RTCV (p = 0.003), lapses (p<0.001)] and end of night vs day shifts [RTM (p = 0.007), RTCV (p = 0.046), lapses (p = 0.001)]. Only lapses were significantly increased at the end of the last (p = 0.013) vs first shift (p = 0.009) in a block of day and night shifts. (2) Duration of sleep before the night (p = 0.019) and consecutive night shifts was decreased significantly (p = 0.034). Subjective sleepiness worsened after day (p = 0.014) and night shifts (p<0.001).

**Conclusion:**

Fatigue worsened after the 12-hour day and night shifts with a greater change after night shifts. Lapses increased after block of day and night shifts. Sleep was decreased before night shifts. Our findings need to be confirmed in larger studies.

## Introduction

Shift work is common in the service industry [1–3], with nearly one-fourth of health employees working as shift workers in the United States [4]. Shift work is associated with a wide range of health problems [5–14]. Fatigue, increased sleepiness, sleep disturbances, and poor cognition are instant effects of shift work [15–17]. This can be explained by the circadian misalignment caused by shift work. The circadian rhythm is controlled by the suprachiasmatic nuclei in the brain and is predominantly regulated by the light/dark cycle [18] to maintain wakefulness and sleepiness rhythm over a ~24-hour period [19]. The natural rhythm promotes arousal in the day and sleepiness in the night which is balanced by two principal forces, the homeostatic sleep pressure and circadian arousal signal [20]. Researchers state that there is a disconnect in this rhythm in shift workers [21]. Night shift workers are awake at night when the circadian arousal signal is at the lowest, and homeostatic sleep pressure is at its peak contributing to sleepiness. In contrast, night shift workers sleep during the day when there is a reversal in these forces which can disturb sleep [21]. Social pressure [22] and individual variability to tolerate shift work are important factors that can further affect circadian misalignment [23]. Sleepiness and fatigue from shift work have implications on work performance and accidents [24]. Health employees working night shifts are prone to make errors while treating patients [16, 25]. There is a higher risk of making errors in night vs day shift and 12-hour vs 8-hour shift [26].

An indirect marker of fatigue is psychomotor vigilance (PV) impairment [27]. Psychomotor vigilance task (PVT) has been used to measure the ability to remain alert and vigilant [28]. Furthermore, it has been validated as a simple and suitable tool to assess the neurocognitive effects of sleepiness and sustained alertness affected by fatigue [29, 30].

Ours is the sole regional level III neonatal referral unit for the state of Western Australia, based at King Edward Memorial Hospital (KEMH) in Perth. It is one of the largest and busiest such neonatal intensive care units (NICU) in the southern hemisphere with 100 beds (Level III: 30, Level II: 70). Neonatal registrars are pivotal in providing care to the critically ill neonates in the unit. Their shift work is rostered in such a way that a registrar is in-house 24 hours a day throughout the year. Their shifts are lengthy (up to 13 hours), and variable (day, night, and on-call for sick cover and neonatal retrievals). Given the size of the unit and its workload, shift work of neonatal registrars in our NICU is mentally and physically demanding. Fatigue and sleepiness related to the demanding shift work are thus significant issues in our unit. We hence aimed to study this issue in our NICU. We hypothesized that the 12-hour rotating shifts will increase the risk of fatigue and affect sleep in registrars working in our NICU.

## Methods and participants

### Design, setting, and ethics

This prospective observational study was conducted in our NICU at KEMH over a period of four weeks. The study protocol was approved by Department of Health, Western Australia—Human Research Ethics Committee (RGS002691).

### Participants

All registrars in KEMH NICU were invited to participate in the study by email advertising and in-service information sessions. All participants gave written informed consent. Consenting registrars were provided envelopes containing a unique participant identity number and questionnaires. The contents of the envelope included the demographic questionnaire, consensus sleep diary (CSD)-core, self-reported sleepiness questionnaire, and additional data form for each shift (as described below). The participant identity number was used to complete the questionnaires and to perform the PVT to protect confidentiality. Based on their request, the personal details of participating registrars were not collected to protect their confidentiality. There were no exclusion criteria.

### Roster

**Fig 1:**
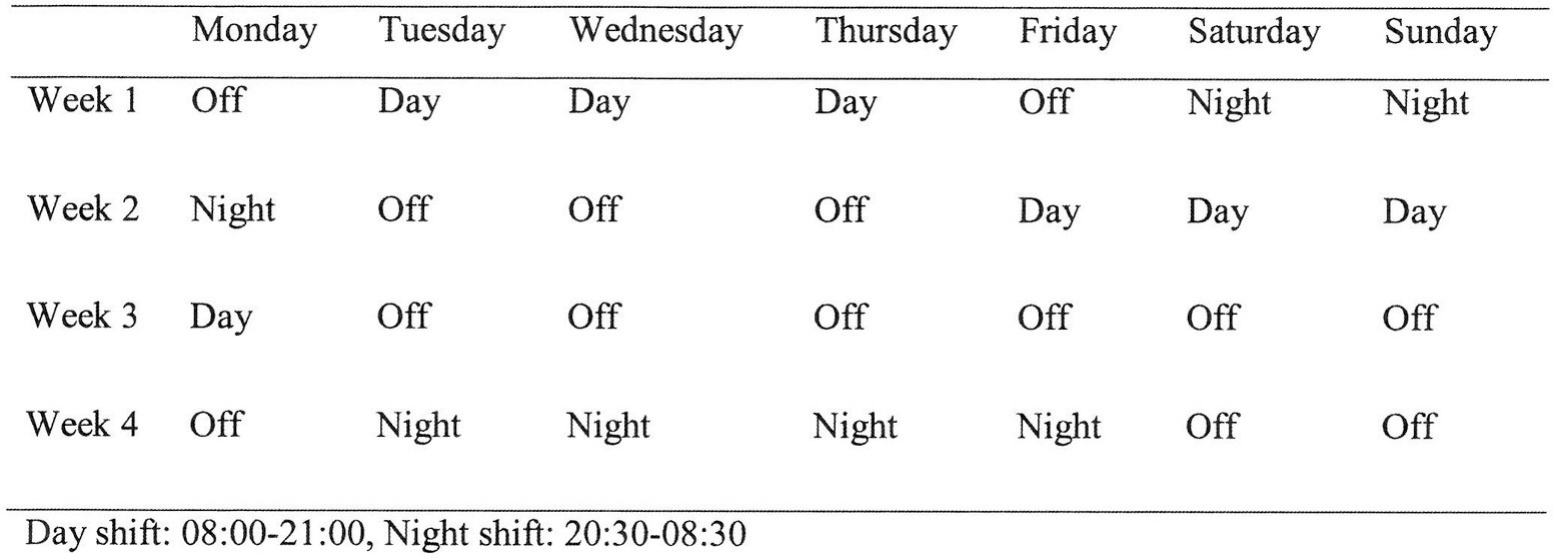
Roster.

The pattern of the roster for registrars during the study period included being in house for 13-hour day (08:00–21:00) and 12-hour night (20:30–08:30) shifts (Fig 1). The expected working hours were 80 hours per fortnight excluding the on calls. This roster resulted in three to four consecutive day or night shifts. The rest period between consecutive shifts was a minimum of one and a maximum of seven days.

### Demographic questionnaire

This included the experience (number of years) as a shift worker and working in a NICU.

### Sleep diary

The CSD-core has been developed by collaboration between insomnia experts [31]. It has 9 questions which include sleep duration and quality (1 = Very poor, 5 = Very good, Range 1–5). Assessment of its quantitative criteria has shown that CSD is a useful screening tool in patients with primary insomnia [32]. Further support for the validity, clinical utility, and usability of CSD was shown in a study that compared CSD indices with similar objective indices and subjective insomnia severity measures between good sleepers and those with insomnia [33]. Participants had to maintain the sleep diary daily.

### Self-reported sleepiness questionnaire

The participants completed a self-reported sleepiness questionnaire “How sleepy are you?” (1 = Not at all, 9 = Very, Range 1–9) before they started the PVT. The self-reported sleepiness questionnaire was used to assess subjective sleepiness.

### PVT

PC-PVT software [Biotechnology HPC Software Applications Institute (BHSAI), MCMR-TT504 Scott Street Ft. Detrick, MD 21702–5012] was used for vigilance testing which is comparable to PVT-192, the gold standard for measuring PV. PC-PVT software has an average delay of <10 milliseconds (ms) in measuring reaction time (RT) which is a margin of error comparable to PVT-192 [34]. The PVT-10 min has been used to measure vigilance and attention [28, 35]. The PC-PVT software was installed on two desktop computers in the office of the neonatal registrars. Each test involved a 10-minute session where visual stimuli appear at variable intervals of 2 to 10 seconds and a mouse button click served as the response. At the end of each PVT session, the data was automatically saved in a subject-specific directory using a text-based file format. The principal investigator was present for all testings to facilitate test completion, monitor compliance, and to prevent external disturbance/distraction to the participant during the testing. PVT was tested at the start and end of each shift. During the PVT, only participants were allowed in the room along with the principal investigator. There was a trial period of two weeks before the start of the study during which participants were made familiar with the software and methodology of testing. Participants were allowed a single training session to minimize learning effects. Mean reaction time (RTM) and relative coefficient of variation (RTCV) were used as primary measures to assess fatigue. RTM was used to index psychomotor speed and RTCV to index lapsing. Secondary measure used to assess fatigue was the number of lapses (RT>500ms).

### Additional data

Additional data was collected to assess the acuity during each shift. This involved questions regarding bed occupancy, new admissions, and neonatal emergency calls (code blue). Participants completed the additional data form at the end of each shift.

All forms were dropped at a secured site in the hospital.

### Outcome measures

#### Primary outcome

**(1)** To compare fatigue at the:(i) “Start vs end” of day and night shifts, (ii) End of “day vs night” shifts, and (iii) End of “first vs last shift” in block of day and night shifts. The RTM, RTCV, and number of lapses were used as markers of fatigue on PVT. **(2)** Duration and quality of sleep before day vs night shifts.

#### Secondary outcome

Subjective sleepiness (self-reported sleepiness questionnaire) at the start vs end of day and night shifts.

### Statistical analysis

Continuous data including demographic, sleep, unit characteristics, and PVT measurements were not normally distributed and were summarised using medians, interquartile ranges (IQR), and ranges (R). Unit characteristics including bed occupancy, new admissions, and emergency calls were compared between day vs night shift using the Mann-Whitney test. Fatigue was measured by PVT responses which included RTM, RTCV, and number of lapses which were transformed to the natural logarithm to correct non-normality to satisfy assumptions for analysis. Comparisons of responses were made using linear regression analysis with a robust variance estimator to address the multiple observations on each participant. When the end of shift “day vs night” and “first vs last shift” responses were analysed as an outcome, the response at the start of the shift and quality of sleep before the shift were adjusted for in the model. The effect of bed occupancy, new admissions, and number of emergency calls on end of shift responses were also considered. For ease of interpretation, start vs end of shift data were presented in their original scale as medians, interquartile ranges (IQR), and ranges (R), and all adjusted comparisons were presented as back transformed estimated marginal means with their 95% confidence intervals (CI). Stata version 12.0 statistical software (StataCorp. 2016, College Station, TX) was used for statistical analysis, and p-values <0.05 were considered statistically significant.

## Results

A total of 15 registrars participated in the study including ten who contributed data for both day and night shift. Of the remaining five, three contributed only for day shifts and two contributed data only for night shifts. 214 PVT measurements were obtained with median (IQR) measurements per participant as 10 (8–20). The median (IQR) years of experience in shift work, working in NICU were 6.5 (6.0–12.3) and 1.5 (0.5–4.1) years respectively.

### Primary outcome

#### (1) Fatigue

(i) “Start vs end” of day and night shifts (Table 1)

**Table 1:** Comparison of sleep status and fatigue: "Start vs end" of day and night shifts.

	Start of shift	End of shift	p-value*
	Median (IQR;R)	Median (IQR;R)	
**Day shift (n = 47)**			
Self-reported sleepiness	4 (2–5;1–9)	6 (3–7;1–9)	0.014
RTM (ms)	288.4 (276.0–323.2;247.9–585.1)	301.9 (285.4–334.9;247.5–597.5)	0.019
RTCV (ms)	24.6 (21.6–34.2;13.9–81.9)	27.1 (20.7–38.8;11.7–106.4)	0.059
Lapses (number of)	1 (0–5;0–50)	2 (0–5;0–51)	0.001
**Night shift (n = 60)**			
Self-reported sleepiness	4 (3–5;1–9)	7 (5–8;1–10)	<0.001
RTM (ms)	291.7 (279.7–322.6;61.5–370.9)	330.1 (298.1–364.1;249.0–477.5)	0.007
RTCV (ms)	25.7 (19.9–41.6;11.7–154.8)	31.5 (22.8–45.1;14.8–232.9)	0.003
Lapses (number of)	1 (0–4;0–14)	5 (2–9;0–35)	<0.001

RTM, mean reaction time; RTCV, relative coefficient of variation; ms, milliseconds; IQR, interquartile range; R, range.

*p-values were derived using regression analysis for timing of shift effects using the F distribution.

**Day shift:** RTM (Median 288.4 vs 301.9, p = 0.019) and number of lapses (Median 1 vs 2, p = 0.001) increased significantly at the end vs start of the shifts.

**Night shift:** RTM (Median 291.7 vs 330.1, p = 0.007), RTCV (Median 25.7 vs 31.5, p = 0.003) and number of lapses (Median 1 vs 5, p <0.001) increased significantly at the end vs start of the shifts.

(ii) End of the “day vs night” shifts (Table 2)

**Table 2:** Comparison of unit characteristics, sleep and fatigue: Day vs Night shift.

	Day shift (n = 47)	Night shift (n = 60)	p-value*
	Median (IQR, R)	Median (IQR, R)	
**Unit characteristics**			
Bed occupancy	40 (21–46;17–52)	23 (20–45;17–52)	0.104
New admissions	2 (1–3;0–5)	1 (0–2;0–4)	0.157
Emergency call	0 (0–1;0–2)	0 (0–1;0–1)	0.914
	**Mean (95% CI)**	**Mean (95% CI)**	**p-value**
**Sleep characteristics**			
Total sleep quality before shift (rated 1–5)	3.2 (2.9–3.5)	2.9 (2.5–3.2)	0.295
Sleep quality before first shift (hrs)	3.0 (2.8–3.2)	3.0 (2.6–3.5)	0.848
Sleep quality before subsequent shifts (2–4 consecutive shifts)	3.4 (2.7–4.1)	2.8 (2.3–3.3)	0.147
Total sleep before shift (hrs)	7.6 (7.2–8.1) Range: 6–11	7.1 (6.2–7.9) Range: 2–15	0.019
Total sleep before first shift (hrs)	7.8 (7.1–8.6) Range: 6–11	8.6 (7.1–10.1) Range: 4–15	0.357
Total sleep before subsequent shifts (2–4 consecutive shifts) (hrs)	7.4 (6.9–7.9) Range: 6–9	6.2 (5.1–7.2) Range: 2–9	0.034
**End of shift PVT measurements**†			
RTM (ms)	308.6 (294.1–323.9)	333.9 (310.7–358.8)	0.007
RTCV (ms)	29.2 (24.8–34.4)	34.5 (29.5–40.3)	0.046
Lapses (number of)	4.1 (2.4–5.8)	7.5 (4.2–10.8)	0.001

RTM, mean reaction time; RTCV, relative coefficient of variation; ms, milliseconds; PVT, psychomotor vigilance task; IQR, interquartile range; R, range; CI, confidence interval.

*p-values for sleep and PVT measurements were derived using regression analysis for timing of shift effects using the F distribution.

†All end of shift PVT measurements are adjusted for the response at start of shift and quality of sleep before shift.

RTM, RTCV, and number of lapses were significantly increased at the end of night vs day shifts. There were no differences in bed occupancy, new admissions, and emergency calls between day and night shifts.

(iii) End of “first vs last shift” in block of day and night shifts (Table 3)

The number of lapses increased at the end of the last vs end of the first shift in a block of day (Median 1.6 vs 2.8, p = 0.013) and night shifts (Mean 3.6 vs 7.3, p = 0.009). There were no significant changes in RTM and RTCV.

**Table 3:** Comparison of fatigue at end of “first vs last shift” in block of day and night shifts (3–4 shifts).

End of shift PVT measurements†	First shift	Last shift	p-value*
	Mean (95% CI)	Mean (95% CI)	
**Day shift (n = 16)**			
RTM (ms)	298.1 (291.8–304.5)	297.7 (278.9–317.8)	0.966
RTCV (ms)	25.0 (18.1–34.4)	27.1 (22.3–32.9)	0.368
Lapses (number of)	1.6 (0.8–2.4)	2.8 (1.7–3.9)	0.013
**Night shift (n = 22)**			
RTM (ms)	320.4 (283.3–362.4)	335.1 (301.3–372.6)	0.301
RTCV (ms)	27.5 (20.0–37.8)	36.2 (28.8–45.6)	0.062
Lapses (number of)	3.6 (2.0–5.3)	7.3 (4.0–10.6)	0.009

RTM, mean reaction time; RTCV, relative coefficient of variation; ms, milliseconds; PVT, psychomotor vigilance task; CI, confidence interval.

*p-values were derived using regression analysis for timing of shift effects using the F distribution.

†All end of shift PVT measurements are adjusted for response at the start of shift.

**(2) Duration and quality of sleep before day vs night shifts (Table 2).** The total duration of sleep (hours) before a shift was higher for the day compared with night shifts: Mean 7.6 hours (95% CI: 7.2–8.1) vs Mean 7.1 hours (95% CI: 6.2–7.9), p = 0.019. However, sleep quality did not differ. The duration and quality of sleep was further studied before the first and subsequent shifts. The duration of sleep remained significantly higher for days compared with nights before consecutive shifts but not before the first shift, while quality of sleep showed no difference.

### Secondary outcome

**Subjective sleepiness (self-reported sleepiness questionnaire) at the start vs end of day and night shifts (Table 1).** Self-reported sleepiness increased from the start to the end of the day (Median 4 vs 6, p = 0.014) and night shifts. (Median 4 vs 7, p<0.001).

## Discussion

The results of our study showed fatigue after 12-hour day and night shifts in registrars doing shift work in our NICU. Sleep was adversely affected before night shifts. Psychomotor speed (RTM) was significantly impaired after both, day and night shifts with a greater impairment after night shifts. The number of lapses had similar results as RTM after day and night shifts and increased in the last compared to first shift while working in a block of day and night shifts. In contrast, RTCV increased only after night shifts. The duration of sleep was significantly reduced before starting night compared with day shift, especially when comparing consecutive shifts compared to first shift. Subjective sleepiness increased after both, day and night shifts. Shift acuity was comparable for day and night shifts. These findings support our hypothesis that 12-hour rotating shifts have adverse effects on our registrars in the NICU.

Sleep before the shift is an important factor to consider. The threshold for the lowest sleep duration to support optimal health in adults as advised by the American Academy of Sleep Medicine and Sleep Research Society is seven hours [36]. Sleep deprivation is known to occur in health workers [37, 38] and shift workers [39]. There was a significant difference in the duration but not quality of sleep before day and night shifts and this was more significant between consecutive night shifts [median (IQR, Range)] [6.2 (5.1–7.2, 2–9)]. This could have contributed to the impaired PV responses after night shifts.

The rationales for selecting RTM, RTCV, and the number of lapses as parameters for fatigue need to be discussed. RTM is used to index performance speed [40, 41]. Performance speed has been recommended as a primary performance index in the neuropsychological perspective [42]. Lapses are continuation of reaction times with extreme results [25] and defined as RT> 500 ms [28, 43–46]. Lapses during PVT are a marker of fatigue [47]. However, it would be inadequate to define a fixed cut off for lapses due to the trade-off between increased performance speed and error frequency. Cumulative distributive function (CDF) analysis is used to define lapses of responding in chronometric tasks where RT is the primary measure of performance [40–42]. RTCV, a measure of reaction-time variability, is an appropriate alternative to CDF to index lapsing or performance instability [40–42].

Our results need to be compared with similar studies reported previously. Ganesan et al. measured PVT (mean RT, fastest 10% of RT, lapses) at the start, middle, and end of the day and night shifts in intensive care unit (ICU) doctors and nurses [39]. PVT responses were more impaired at the end of the night compared to day shifts and worsened in the middle and end of night shift as compared to the start [39]. Bihari et al. studied sleepiness and alertness in medical officers working in the ICU and found that PVT changes (median response times and lapses) increased after night shifts [48]. Nurses on rotating shifts have been shown to have reduced vigilance (mean reaction time, lapses) after night vs day shifts [27]. It is important to note the variance in shift duration between our study and the above mentioned studies. We found an increase in RTM and lapses at the end vs start of the day and night shifts and end of night vs day shift. RTCV was significantly increased at the end vs start of night shifts and end of night vs end of day shift. These results suggest impairment in vigilance after 12-hour day and night shifts with more attentional fluctuations after night shifts. PVT studies in health professionals working fixed shift blocks (nights or days) have demonstrated neurocognitive impairment after night shifts [49–51]. Shift workers working night shifts face circadian misalignment and increasing homeostatic sleep pressure which could explain the worsening of subjective and objective measures of sleepiness [52].

The limitation in the assessment of subjective sleepiness in our study needs to be accepted. We used a self–reported sleepiness questionnaire which has not been validated as compared to the Dundee Stress State Questionnaire (DSSQ) and Karolinska Sleepiness Scale (KSS) which are validated tools. The DSSQ is well evaluated to assess the fundamental dimensions of subjective state in a performance setting [53, 54]. The KSS is used to measure subjective sleepiness. Our scale is similar but not identical to KSS. It is sensitive for detecting sleep deprivation and correlates with physiological and behavioural indicators of sleepiness and performance [55–57]. Scores ≥7 on KSS have been reported to correlate with electroencephalographic signals of objective sleepiness [57]. Two studies in health workers have shown a significant worsening of KSS scores at the end compared to the start of the night shift [39, 48]. Furthermore, one of them showed significant change after a day shift [48]. The median score for self–reported sleepiness in our study increased from 4 and 4 at the start to 6 and 7 at the end of day and night, respectively. These results suggest that subjective sleepiness worsened after day and night shifts, and the change is greater after night shifts.

Appreciating the significance of our results related to consecutive night shifts is important. Our registrars worked in a block of 3 to 4 shifts. We found a significant increase in the number of lapses at the end of the last vs first shift in both, day and night shifts. Importantly, the lapses were more frequent in the night compared to day shifts. Ganesan et al. compared PVT responses on consecutive night shifts and found no difference in mean RT, lapses, and fastest 10% of RT between the first and last night shift (up to 5 shifts) [39]. Behrens et al. reported that nurses working on consecutive day and night shifts had improved performance after the third night shift. Training effect and better adaptation to the night shifts were suggested as the possible reasons for these findings [27]. Cumulative sleep deprivation can occur while working consecutive shifts due to inadequate sleep accumulating after each shift. However, sufficient sleep can mitigate the effects of cumulative sleep deprivation derived from working several night shifts [35]. Registrars in our study had decreased duration of sleep especially between consecutive night shifts which could have contributed to the increase in the number of lapses at the end of the night shifts. RTM and RTCV increased at the end of the last vs first night shift compared to day shifts but didn’t reach statistical significance. It is difficult to comment whether adaptation has a role to play in these findings.

Frequent alternating between different shift blocks has been shown to have a significant effect on the duration of sleep and wake time between shifts in intensive care workers [39]. Registrars in our unit worked in blocks of 3–4 shifts and had rest periods between 1 to 7 days. However, they would be on call for sick cover and neonatal retrievals on their days off. These factors could have impacted their sleep and fatigue. We are unable to comment on this issue in the absence of specific data on activities during the “off-call” period.

Other limitations of our study need to be discussed. Naps during shift [58, 59], sleep inertia [60], and intake of caffeinated drinks [61, 62] can impact sleep and fatigue. Napping during shifts has been linked to improvement in subjective and objective measures of alertness [63–65]. Our registrars could have naps during night shifts if there were no acute issues. However, in absence of data; we are unable to comment on the effect of these variables on our results. Our study was conducted in a small and select population at a single institution, which prevents the generalization of our findings. We did not obtain baseline demographic parameters to protect the confidentiality of the participants. Furthermore, we didn’t use an actigraph which is a validated and reliable instrument to measure the circadian rhythm, pattern of rest, mobility, and the sleep-wake cycle.

## Conclusion

In summary, the results of our study showed that working 12-hour rotating shifts adversely affected sleep and increased the risk of fatigue in our registrars. The adverse effects were more significant after night shifts. Lapses increased after block of day and night shifts. The significance of our findings need to be confirmed in larger studies.

## Supporting information

S1 Fig Consensus sleep diary–Core. (TIF)

S2 Fig Self-reported sleepiness questionnaire. (TIF)

S3 Fig Additional data form. (TIF)
