# Supplementary figures and images for: Effect of shift work on fatigue and sleep in neonatal registrars
Source: PLoS One. 2021 Jan 14;16(1):e0245428. doi: 10.1371/journal.pone.0245428 (PMC7808639; doi:10.1371/journal.pone.0245428)

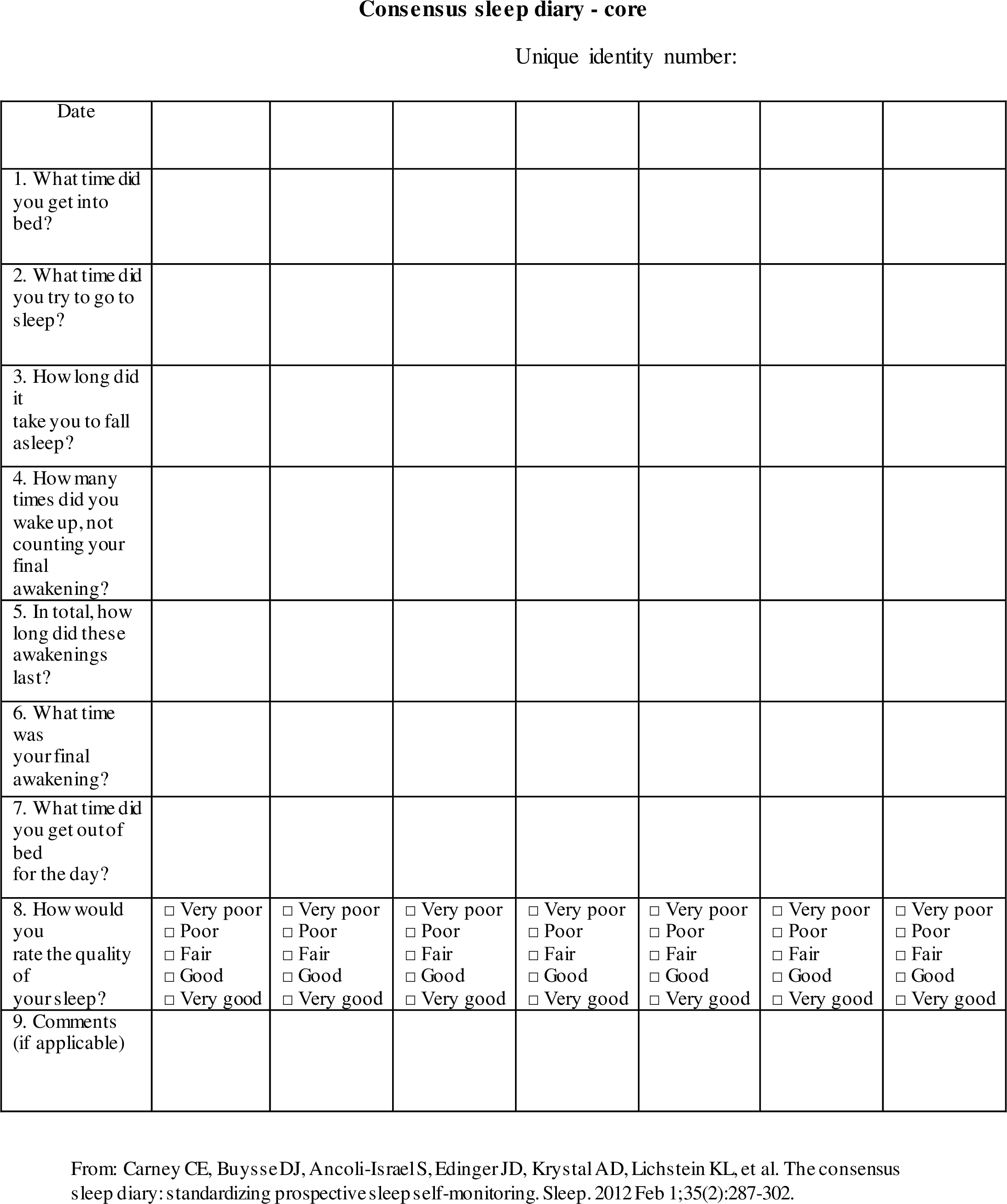

Supplement: S1 Fig — (TIF) [file pone.0245428.s001.tif]

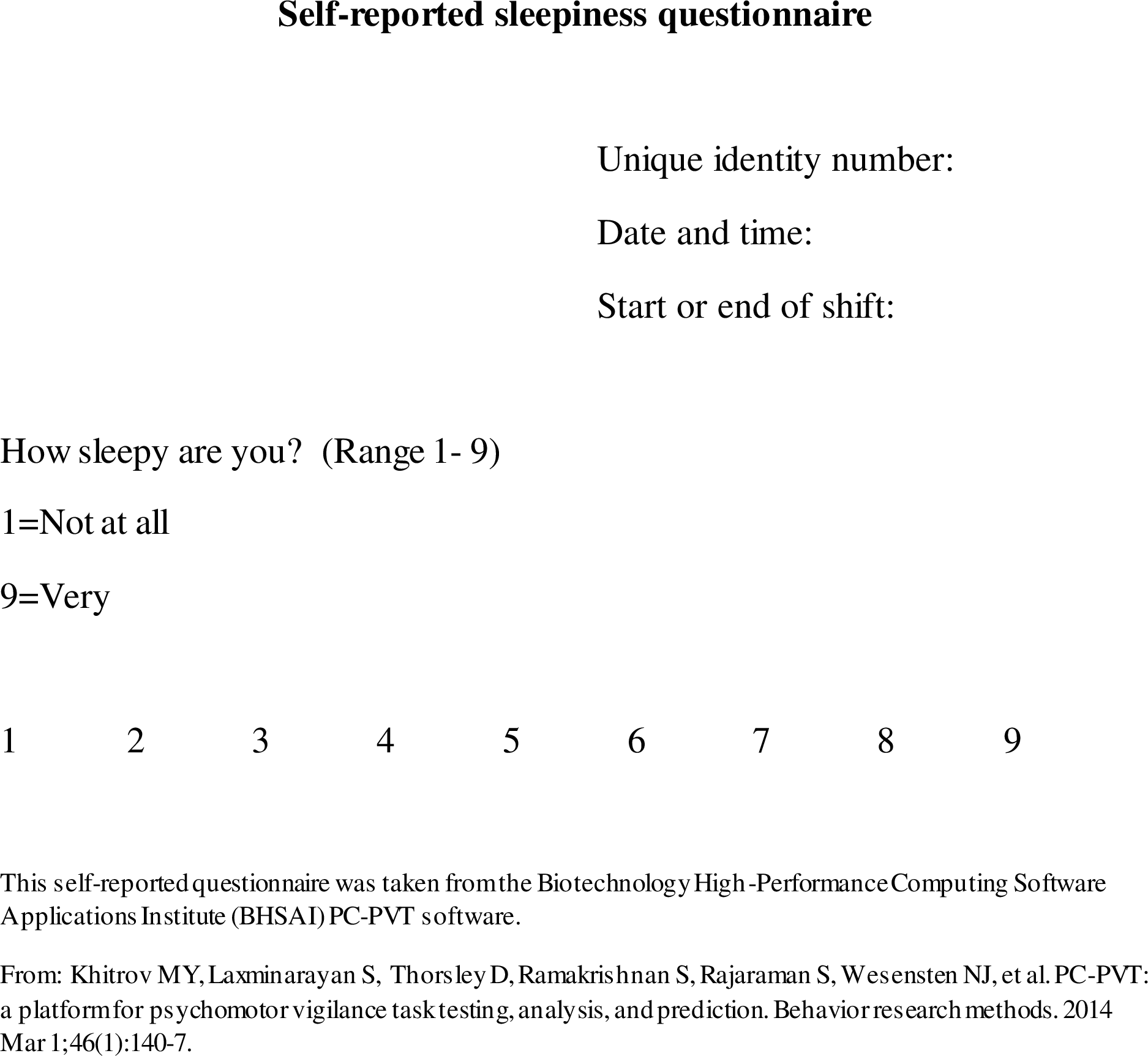

Supplement: S2 Fig — (TIF) [file pone.0245428.s002.tif]

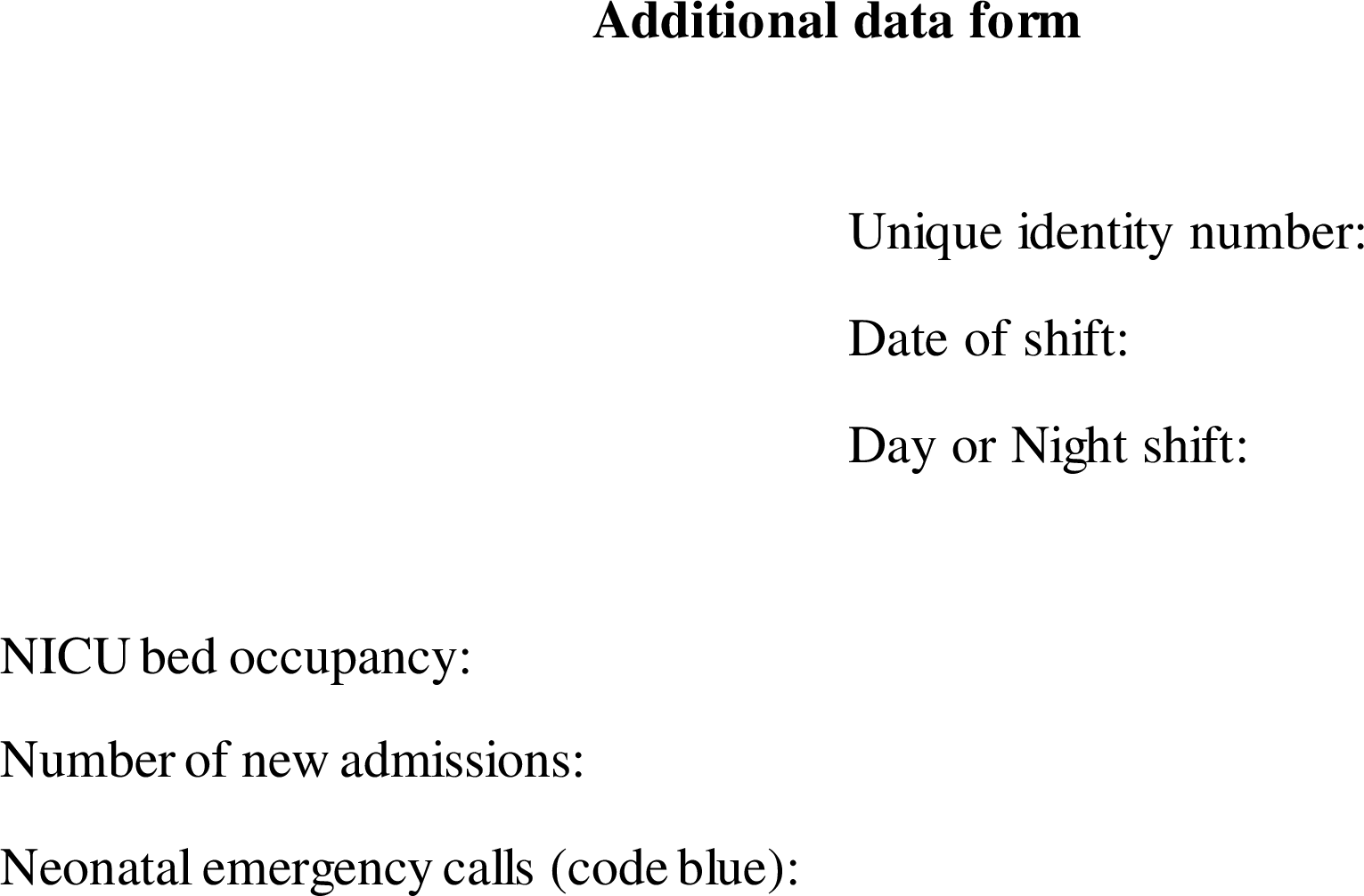

Supplement: S3 Fig — (TIF) [file pone.0245428.s003.tif]
